# Multi-Scale Synergistic Mechanism of Damping Performance in Crumb Rubber-Modified Asphalt

**DOI:** 10.3390/polym18010090

**Published:** 2025-12-28

**Authors:** Wenqi Kou, Mingxing Gao, Ting Zhao, Danlan Li, Hangtian Li

**Affiliations:** College of Energy and Transportation Engineering, Inner Mongolia Agricultural University, Hohhot 010018, China; kouwenqi@imau.edu.cn (W.K.); zhaoting666@imau.edu.cn (T.Z.); ldlnyjt@imau.edu.cn (D.L.); lihangtian@imau.edu.cn (H.L.)

**Keywords:** crumb rubber-modified asphalt (CRMA), damping performance, multi-scale analysis

## Abstract

Utilizing waste tire crumb rubber to modify asphalt enhances the damping and noise reduction performance of pavements. This study employs a multi-scale approach to investigate the effect of crumb rubber content (5–25%) on the damping performance of crumb rubber-modified asphalt (CRMA). The results show that damping performance improves initially with increasing crumb rubber content, peaking at 20%, and then declines. At this optimal content, the loss modulus increases by 110% and 440% at 46 °C and 82 °C, respectively, compared to base asphalt, with enhanced damping efficiency and damping temperature stability. Fluorescence microscopy (FM) images and quantitative analysis reveal that, at 20%, the crumb rubber forms a moderately connected three-dimensional network. Molecular dynamics (MD) simulations indicate that, at this content, the solubility parameter of the CRMA system is closest to that of the base asphalt, and interfacial binding energy increases, suggesting optimal compatibility. Ridge regression models, with R^2^ values of 0.903 and 0.876 for the FM and MD scales, respectively, confirm that crumb rubber dispersion is the dominant factor governing damping performance, with moderate phase separation further enhancing performance. This study establishes a quantitative structure–property relationship, providing a framework for understanding the damping performance of rubber-modified asphalt pavements.

## 1. Introduction

With the continuous growth of global vehicle ownership, the total number of vehicles is expected to exceed 2 billion by 2030, exacerbating the challenges of waste tire accumulation and traffic noise pollution [1]. Each year, approximately 1.3 to 1.5 billion waste tires are generated globally, weighing a total of 166 million tons. Improper disposal of these tires poses significant threats to environmental integrity and public health [2]. Moreover, traffic noise accounts for approximately 70% of total environmental noise, and its continued intensification poses substantial risks to the physical and mental health of urban populations [3,4,5]. Consequently, integrated management of these two pressing environmental issues has become increasingly urgent [6]. In this context, crumb rubber-modified asphalt (CRMA) pavement, which integrates the resource utilization potential of waste tires with noise reduction capabilities, has attracted considerable attention. Asphalt pavement is widely recognized as one of the most effective solutions for mitigating traffic noise, owing to its superior damping and noise reduction performance [7,8]. The incorporation of crumb rubber further enhances both damping and noise reduction while simultaneously facilitating the large-scale consumption of waste tires. This approach offers a promising technological solution for the integrated management of waste tire accumulation and traffic noise reduction [9,10,11,12,13].

Owing to the favorable damping characteristics of crumb rubber, rubberized mixtures prepared using the dry process have demonstrated promising potential for road noise reduction and have been extensively studied. Biligiri et al. [14] compared the damping parameters of 159 conventional dense-graded asphalt mixtures with 9 rubberized mixtures and found that the incorporation of crumb rubber notably enhanced the damping response of the asphalt mixtures. Quan et al. [15] investigated the influence of crumb rubber content and particle size on the damping performance of rubberized mixtures, revealing that as the crumb rubber content increased, the damping ratio of the mixture also progressively improved. Kehagia and Mavridou [16] conducted field tests and observed that conventional asphalt pavements produced noise levels ranging from 71 to 73 dB, while rubberized mixtures achieved noise levels between 68 and 70 dB, resulting in a noise reduction of approximately 1 to 5 dB. Jin et al. [17] further substantiated these findings through systematic noise testing, confirming that rubberized mixtures consistently reduced noise by 2 to 3 dB under varying driving speed conditions. Additionally, Wu et al. [3] combined tire vibration and acoustic tests to demonstrate that the incorporation of crumb rubber significantly enhanced both the damping performance and sound absorption coefficient of asphalt mixtures, thereby improving the overall noise reduction effectiveness of the tire-pavement system. In conclusion, numerous studies have consistently demonstrated the effectiveness of rubberized mixtures in enhancing pavement damping and noise reduction performance.

However, the damping in asphalt primarily arises from the viscoelastic properties of the asphalt binder or polymer modifiers, rather than from rigid fillers or aggregates [9]. Therefore, investigating the properties of the asphalt binder is crucial to understanding the enhanced damping performance of asphalt pavement. Jiang et al. [6,9] investigated the damping mechanism of CRMA by combining damping-temperature spectra with microscopic techniques. Xu et al. [18] explored the impact of aging on the damping characteristics of CRMA using damping-temperature spectra and FTIR analysis. Despite these efforts, existing studies have not fully explored the relationship between macroscopic performance and microscopic structure, leaving a systematic cross-scale linkage insufficiently developed.

Asphalt is inherently a heterogeneous organic material with a complex molecular structure, and the incorporation of crumb rubber further increases the complexity of the system. These characteristics pose significant challenges in systematically elucidating the evolution of asphalt’s damping mechanism through experimental methods alone. Additionally, the intermolecular interactions that influence the macroscopic properties of asphalt materials are difficult to directly capture using conventional testing techniques [19]. To address these limitations, molecular dynamics (MD) simulation, as an advanced technique operating at the molecular scale, enables the direct observation and quantification of intermolecular interactions between asphalt components and crumb rubber within physically realistic virtual models. This approach overcomes the limitations of conventional testing methods and experimental preparation, and has been increasingly applied to studies on the rheological properties, aging behavior, and other characteristics of CRMA [20,21,22]. However, research focused on systematically clarifying the damping and noise reduction mechanisms of CRMA by quantitatively linking molecular-scale interaction parameters with macroscopic performance indicators remains relatively limited.

Building upon this, a multi-scale collaborative analysis framework was established in this study, spanning the macroscopic, microscopic, and molecular scales, to systematically elucidate the influence mechanism of crumb rubber content on the damping performance of CRMA. Through dynamic shear rheometer (DSR) tests, fluorescence microscopy (FM) observations, and molecular dynamics (MD) simulations, the macroscopic damping performance, microscopic dispersion state, and intermolecular interaction parameters of CRMA were obtained, respectively. Subsequently, gray relational analysis and ridge regression methods were applied to correlate and model the multi-scale parameters, thereby constructing a quantifiable multi-scale coordination equation. This approach not only provides a quantitative mechanistic interpretation of the relationship between macroscopic performance and molecular parameters but also systematically reveals the cross-scale synergistic mechanism underlying optimal damping performance, viewed from the perspectives of microstructural evolution and molecular interactions.

## 2. Materials and Methods

### 2.1. Materials

The base asphalt used in this study is 90# Karamay asphalt. The crumb rubber modifier, consisting of 80-mesh desulfurized crumb rubber, was supplied by Fenyang Ruifeng Rubber Co., Ltd. (Fenyang, China). The crumb rubber was produced through a thermochemical treatment process, in which tire-derived crumb rubber was reacted with a proprietary devulcanizing agent in a sealed tank at 180 °C for 30 min. This process disrupts the cross-linking bonds and network structure present in crumb rubber, thereby enhancing the surface activity of the desulfurized crumb rubber [23]. The basic characteristics of the base asphalt and crumb rubber, as specified by the relevant Chinese standard specifications and technical guidelines, are summarized in Table 1, which also provides other key information about the materials and ensures compliance with all specification requirements.

### 2.2. Preparation of Specimens

The CRMA was prepared in accordance with relevant Chinese industry standards and specifications [24,26]. Initially, the base asphalt was placed in an oven at 135 °C for 1 h until fully melted, reducing its viscosity for subsequent processing. It was then transferred to a high-shear mixer (Fluko, Shanghai, China) preheated to 140 °C and sheared at 3000 rpm to improve the uniformity of the base asphalt. Subsequently, the temperature was raised to 180–185 °C, and crumb rubber was added at predetermined proportions (5%, 10%, 15%, 20%, and 25% by mass of base asphalt). Each batch of crumb rubber was added within 1 min, followed by high-speed shearing at 5000 rpm for 60 min within this temperature range. This stage represents a critical process in which the crumb rubber undergoes swelling, degradation, and modification reactions with asphalt under high-temperature conditions [27,28]. Finally, the prepared CRMA was placed in an oven at 140 °C and allowed to stand for 30 min until all bubbles disappeared, ensuring proper curing. The resulting samples were then transferred into airtight, light-proof containers and stored under appropriate conditions. All subsequent tests were conducted sequentially within 24 h of preparation [29].

### 2.3. Experimental Section

#### 2.3.1. Dynamic Shear Rheometer (DSR) Test

In dynamic mechanical analysis, the damping characteristics of asphalt binders are comprehensively evaluated from two perspectives: energy dissipation capacity and energy dissipation efficiency [30]. The loss modulus directly represents the energy density dissipated by the material due to viscous deformation under cyclic loading, serving as a key indicator of its intrinsic energy dissipation potential. The loss factor, defined as the ratio of the loss modulus to the storage modulus, reflects the viscoelastic balance and energy dissipation efficiency of the material. Jiang et al. [6,9] established an evaluation criterion for the energy dissipation efficiency of asphalt binders by constructing a damping-temperature spectrum, with the loss factor plotted against temperature. This criterion, based on the temperature-dependent stability of the loss factor, comprises two key indicators: (1) a high peak value and (2) a broad integrated area under the damping-temperature spectrum. For asphalt pavement applications focused on damping and noise reduction, the binder must exhibit both a high loss modulus to dissipate more energy and an appropriate loss factor to ensure stable viscoelastic behavior and efficient energy dissipation across a wide temperature range. Therefore, this study systematically evaluates the damping performance of CRMA by integrating both the loss modulus and the damping-temperature spectrum.

To determine the loss modulus and loss factor of CRMA at various temperatures, temperature sweep tests were performed using a DSR on a Discovery HR-1 rheometer (TA Instruments, New Castle, DE, USA) [18]. These tests were conducted in accordance with the AASHTO T 315 standard [31] and Superpave performance specifications [32], considering typical pavement service conditions and the requirements for damping characterization. The following test parameters were applied: (1) a fixed angular frequency of 10 rad/s to simulate typical shear loading rates at constant vehicle speeds; (2) a temperature sweep from 46 °C to 82 °C in 6 °C intervals, encompassing both normal and elevated pavement service temperatures to capture the trend of damping performance with temperature; and (3) all tests were carried out in strain-controlled mode with a constant strain amplitude of 0.1%, verified through preliminary amplitude sweep tests to ensure that measurements remained within the linear viscoelastic range, thereby providing intrinsic property data. Each test was performed in triplicate, and the average result was used for analysis.

#### 2.3.2. Fluorescence Microscopy (FM) Test

Microstructural characterization was conducted using a Leica DM2700M upright metallurgical microscope system (Leica Microsystems, Wetzlar, Germany), equipped with a 50 W high-pressure mercury lamp and coupled with an EBQ 100-04 precision light intensity control device. By exploiting the characteristic yellow-green fluorescence of crumb rubber under blue/UV excitation (excitation filter: BP 450–490 nm bandpass, emission filter: LP 515 nm longpass), all CRMA samples were simultaneously imaged in both bright-field and fluorescence modes at 100× magnification (numerical aperture NA = 0.9) to ensure spatial consistency between the morphological features and fluorescence signals.

**Figure 1:**
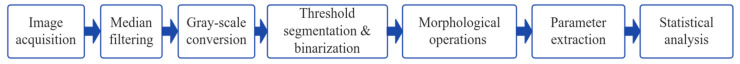
Flowchart for FM image processing and analysis. The arrows indicate the sequential workflow of the image processing steps.

To objectively quantify the microscopic dispersion of crumb rubber in asphalt, FM images were processed and analyzed using Image Pro Plus software (Version 6.0), following the procedure outlined in Figure 1 [33,34]. First, the collected brightfield and fluorescence images were aligned. Median filtering was then applied to reduce noise, followed by grayscale conversion. Image segmentation was performed using a fixed-threshold binarization method, with the threshold value set to a grayscale level of 140, determined based on the typical image histogram and preliminary experimental results. After binarization, morphological operations were applied to refine the target contours, and feature phases were extracted. To ensure data accuracy, preliminary experiments confirmed that five non-overlapping fields of view from representative areas were randomly selected for image acquisition and analysis per sample. The final experimental parameters were calculated as the average of the statistical results from these five fields of view.

**Figure 2:**
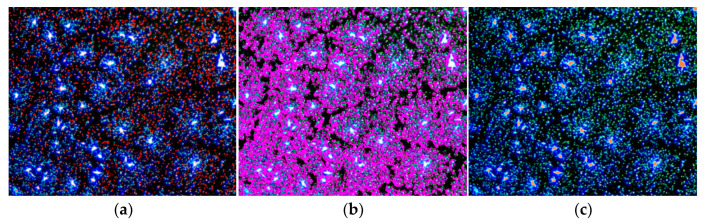
Three characteristic phases were identified in the FM images. The dark background corresponds to the non-fluorescent base asphalt, while the bright regions are attributed to crumb rubber exhibiting characteristic fluorescence under the specified excitation filters. (**a**) Dispersed crumb rubber, identified by red markers; (**b**) Gel-type transition zone, identified by magenta markers; (**c**) Crumb rubber agglomeration phase.

The image processing procedure aims to characterize three distinct phases: dispersed crumb rubber, the gel-type transition zone, and the crumb rubber agglomeration phase. Schematic representations of these phases are shown in Figure 2, and the corresponding parameters are calculated as follows:

Rubber Dispersion Degree (RDD): RDD is defined as the area percentage of the dispersed rubber phase (including both crumb rubber and swollen colloid) relative to the total effective area of the analyzed image. The calculation formula is:

(1) $$RDD=\frac{A_{phase}}{A_{total,pixels}}\times 100\%$$

where *A*_phase_ is the total pixel area of the fluorescent bright domain (crumb rubber/colloid), and *A*_total,pixels_ is the total pixel area of the effective image region designated for analysis.

Relative Area of Colloid Domain (RACD): RACD quantifies the relative area occupied by the gel-like interfacial transition zone, which exhibits uniform morphology after the swelling of crumb rubber. This domain is distinguished from the total fluorescent area through specific morphological operations. The calculation formula is:

(2) $$RACD=\frac{A_{colloid}}{A_{total,pixels}}\times 100\%$$

where *A*_colloid_ is the pixel area identified as the colloid domain after morphological processing.

Number of Agglomerates per Unit Area (NA): NA quantifies the number density of distinct rubber agglomerates. Agglomerates are defined as connected regions with continuous fluorescent pixels exceeding a size threshold. The calculation formula is:

(3) $$NA=\frac{N_{agglomerates}}{A_{total,field}}$$

where *N*_agglomerates_ is the number of connected regions meeting the size threshold, and *A*_total,field_ is the total field area (typically in mm^2^).

### 2.4. Molecular Dynamics (MD) Simulation

#### 2.4.1. Molecular Composition of Base Asphalt

Molecular dynamics models for asphalt were constructed based on the widely adopted four-component framework. The molecular representations of the components are as follows: asphaltenes were modeled using the Mullins continental model; aromatics were represented by 1,7-dimethylnaphthalene (C_12_H_12_); saturates were represented by docosane (C_22_H_46_); and resins were modeled as a polycyclic aromatic hydrocarbon structure [35,36,37].

Based on prior research [38], which characterized the components of 90# Karamay asphalt, we constructed the asphalt molecular model using the Amorphous Cell module in Materials Studio software (Version 2020). The molecular count and composition ratios of the base asphalt are summarized in Table 2.

#### 2.4.2. Molecular Composition of Crumb Rubber

**Figure 3:**
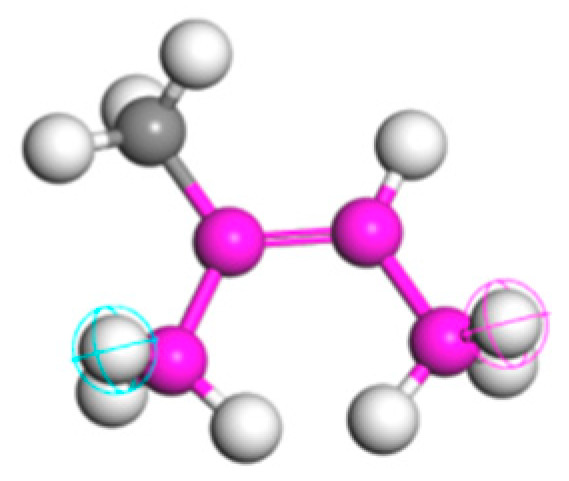
Repeating units of NR. Magenta, gray, and white beads represent main-chain carbons, side-group carbons, and hydrogen atoms, respectively. The chain head and tail are highlighted with light blue and pink crossed circles.

The crumb rubber modifier primarily consists of natural rubber (NR) and styrene-butadiene rubber (SBR), which form the basis for the molecular modeling. As shown in Figure 3, NR is modeled as a homopolymer chain composed of repeating monomer units.

**Figure 4:**
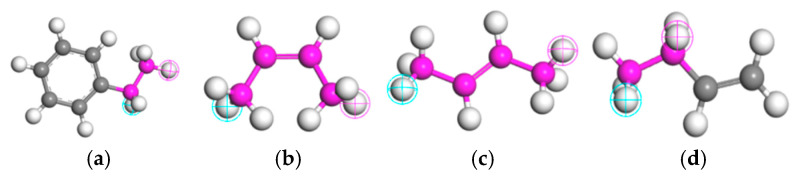
SBR monomers of each molecule. (**a**) Styrene; (**b**) cis-1,4-butadiene; (**c**) trans-1,4-butadien; (**d**) 1,2-butadiene.

As shown in Figure 4, SBR was modeled as a random copolymer consisting of 23.5% styrene and 76.5% butadiene by mass. The butadiene component comprises three isomeric forms by mass: trans-1,4 (76%), cis-1,4 (7%), and 1,2-butadiene (16%), along with minor additional components (1%) [22,39].

During the molecular modeling of the crumb rubber component, the mass ratio of NR to SBR was set at 3:7. The NR and SBR chains were represented by oligomers with polymerization degrees of 3 and 15, respectively [40]. In modeling CRMA with different crumb rubber contents, the molecular structure and quantity of the asphalt component were kept constant. The total number of crumb rubber polymer chains required to achieve each target crumb rubber content was calculated based on the mass of the base asphalt. The corresponding quantities are provided in Table 3.

#### 2.4.3. Construction of MD Models

Based on the molecular compositions presented in Table 2 and Table 3, molecular models for base asphalt, crumb rubber, and CRMA with different crumb rubber contents were constructed. Since the initial molecular models exhibited high energy and structurally unstable configurations, geometry optimization and molecular dynamics simulations were employed to minimize and equilibrate the systems, thereby achieving realistic equilibrium structures. All calculations were performed using the Condensed-phase Optimized Molecular Potentials for Atomistic Simulation Studies (COMPASS) force field. This force field is an optimized ab initio approach, suitable for predicting the properties of common organic and polymeric materials across a wide range of temperatures and pressures [39]. It has been extensively used in simulations exploring the compatibility, microstructure, and mechanical properties of CRMA [38,41,42]. During the simulations, electrostatic interactions were calculated using the Ewald summation method, while van der Waals forces were treated with an atom-based cutoff. Temperature and pressure were controlled using the Nose-Hoover thermostat and the Berendsen barostat, respectively. All calculations were conducted within the Forcite module, following the specific workflow outlined below.

First, geometry optimization was carried out using the “Smart” algorithm, with a maximum of 10,000 iterations. Subsequently, five cycles of simulated annealing were performed under the NVT ensemble, with the temperature ranging from 300 K to 500 K, to eliminate unrealistic molecular conformations. Following this, an equilibrium molecular dynamics simulation was conducted under the NPT ensemble at 298 K and 1 atm. The simulation ran for 200,000 steps (equivalent to 200 ps) with a time step of 1 fs, and conformations were saved every 1000 steps.

**Figure 5:**
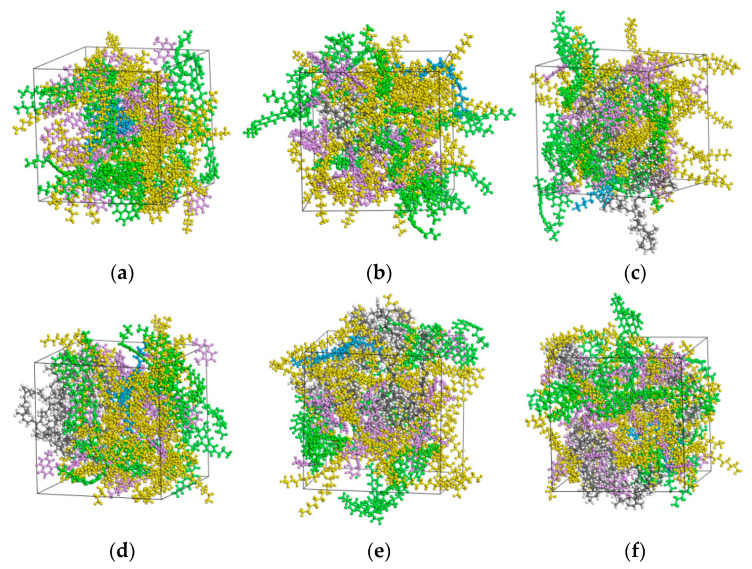
Asphalt binder molecular models. (**a**) Base asphalt; (**b**) 5% CRMA; (**c**) 10% CRMA; (**d**) 15% CRMA; (**e**) 20% CRMA; (**f**) 25% CRMA, where blue represents asphaltenes, pink represents aromatics, green represents resins, yellow represents saturates, and gray represents crumb rubber.

Following the established workflow, molecular models of base asphalt and crumb rubber were constructed, each with an initial system density of 1 g/cm^3^. After geometry optimization and equilibrium simulations, the models attained densities of 0.991 g/cm^3^ and 1.07 g/cm^3^, respectively, which are in reasonable agreement with the corresponding experimental values of 1.02 g/cm^3^ and 1.17 g/cm^3^. These results validate the accuracy of the individual component models. The optimized single-component models were then combined to create initial composite models of CRMA at the target crumb rubber contents. The same comprehensive workflow, including geometry optimization followed by NPT ensemble equilibration, was applied to these composite systems to ensure energetic stability and density convergence. Representative stable conformations were extracted from the equilibrated portion of each trajectory for the calculation of cohesive energy density and binding energy. The resulting equilibrium structures of the base asphalt and CRMA models are shown Figure 5.

## 3. Results and Discussion

### 3.1. DSR Test Results

**Figure 6:**
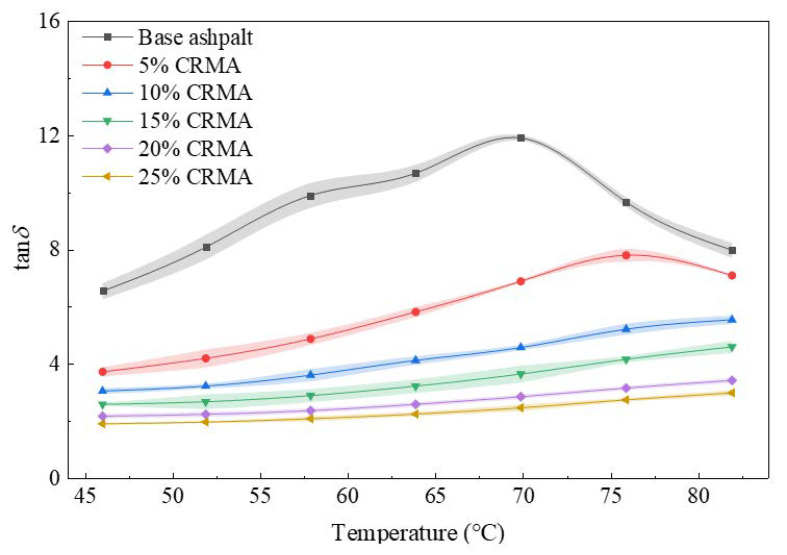
Damping-temperature spectra (tan *δ* vs. temperature) of CRMA at different crumb rubber contents. The shaded regions denote the error bands (standard deviation) based on multiple tests.

**Figure 7:**
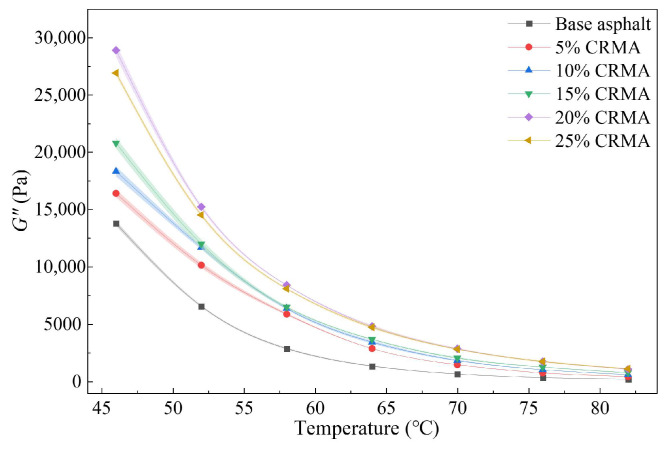
Loss modulus versus temperature for CRMA at different crumb rubber contents.

Temperature sweep tests were conducted using a DSR to measure the loss factor (tan *δ*) and loss modulus (*G*″) of the asphalt specimens. The resulting damping-temperature spectra and loss modulus curves are shown in Figure 6 and Figure 7, respectively. The damping characteristics of the CRMA were evaluated in terms of both damping efficiency and energy dissipation capacity.

As shown in Figure 6, the damping-temperature spectra of CRMA with different crumb rubber contents are generally flatter than that of the base asphalt, indicating that the incorporation of crumb rubber effectively reduces the temperature sensitivity of the binder’s damping properties. However, the extent of this improvement varies significantly with crumb rubber content. For low-content CRMA (5%, 10%, and 15%), the spectrum for 5% CRMA exhibits a distinct inflection point, while for 10% and 15% CRMA, the loss factor increases substantially by 82.0% and 77.2%, respectively, between 46 °C and 82 °C, resulting in steeper curves. In contrast, the damping spectra of 20% and 25% CRMA are notably flatter, reflecting enhanced damping stability across the entire temperature range. Due to this flatness, calculating the integrated area under the curve becomes difficult; therefore, damping efficiency was assessed by directly comparing the loss factor values. Throughout the studied temperature range, the loss factor of 20% CRMA consistently exceeds that of 25% CRMA, indicating superior damping efficiency. Moreover, the non-overlapping 95% confidence intervals confirm that this difference is statistically significant. Therefore, 20% CRMA exhibits optimal damping efficiency.

As shown in Figure 7, the loss modulus of all asphalt specimens decreases sharply with increasing temperature, indicating that their energy dissipation capacity is highly temperature-dependent. The incorporation of crumb rubber significantly enhances the loss modulus of the asphalt. At each temperature, the loss modulus increases with crumb rubber content, initially rising sharply before decreasing, with the peak reached at 20% content. For instance, at 46 °C, the loss modulus of CRMA with 5% to 25% crumb rubber content increased by 19.2%, 33.1%, 51.2%, 110.1%, and 95.7%, respectively, compared to the base asphalt. The confidence intervals for different contents do not overlap, indicating statistically significant differences. After reaching 20% content, the increase in loss modulus slows, and the loss modulus of 25% CRMA is even slightly lower than that of 20% CRMA, reflecting the optimal energy dissipation capability at 20% content. This advantage is observed over a wide temperature range: at 82 °C, the loss modulus of 20% CRMA (1051.16 Pa) is approximately 440% higher than that of the base asphalt (194.71 Pa), confirming that 20% CRMA consistently exhibits superior energy dissipation potential across the entire temperature range.

**Figure 8:**
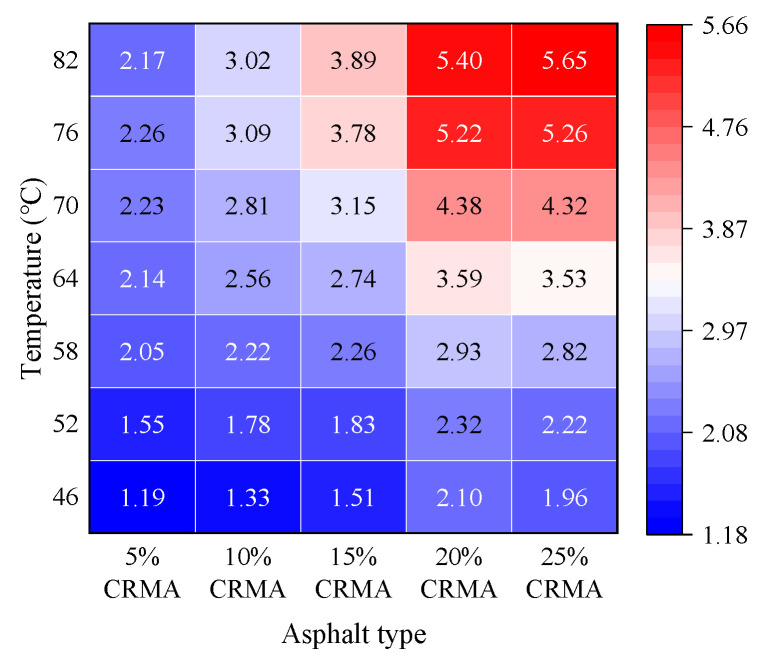
Normalized loss modulus ratio of CRMA to base asphalt versus temperature. Text color (black/white) is auto-adjusted for readability against the cell background.

To eliminate the influence of the base asphalt’s inherent properties and focus on the effect of crumb rubber content on the loss modulus of CRMA, a normalization analysis was performed. This was achieved by dividing the average loss modulus of CRMA specimens with different crumb rubber contents at each temperature by the corresponding average loss modulus of the base asphalt. The resulting normalized loss modulus ratio for the CRMA is presented in Figure 8.

As shown in Figure 8, within the temperature range of 46–82 °C, the normalized loss modulus of CRMA generally increases with higher crumb rubber content. Among all the tested contents, the 20% crumb rubber content exhibits the most significant and stable enhancement across the entire temperature range. For instance, at 82 °C, the average normalized loss modulus of 20% CRMA is 5.40, with a 95% confidence interval of 5.11–5.69 and a coefficient of variation as low as 2.14%, indicating highly concentrated data and reliable results. In comparison, the average normalized loss modulus of 5% CRMA at 82 °C is 2.17, making the former 2.49 times greater than the latter. Moreover, the confidence intervals for these two values do not overlap, demonstrating a highly statistically significant difference. This further confirms the clear advantage of the 20% content at high temperatures from a normalized perspective. The normalization analysis also highlights the synergistic effect between crumb rubber content and temperature. When the temperature exceeds 64 °C, specimens with 15–25% crumb rubber content show significant performance improvements. However, once the content surpasses the critical threshold of 20%, the rate of improvement begins to level off. For example, at 82 °C, although the average normalized loss modulus of 25% CRMA (5.65) slightly exceeds that of 20% CRMA (5.40), the 95% confidence intervals (25%: 5.15–6.16; 20%: 5.11–5.69) overlap partially, indicating that this marginal increase is not statistically significant.

### 3.2. FM Test Results

**Figure 9:**
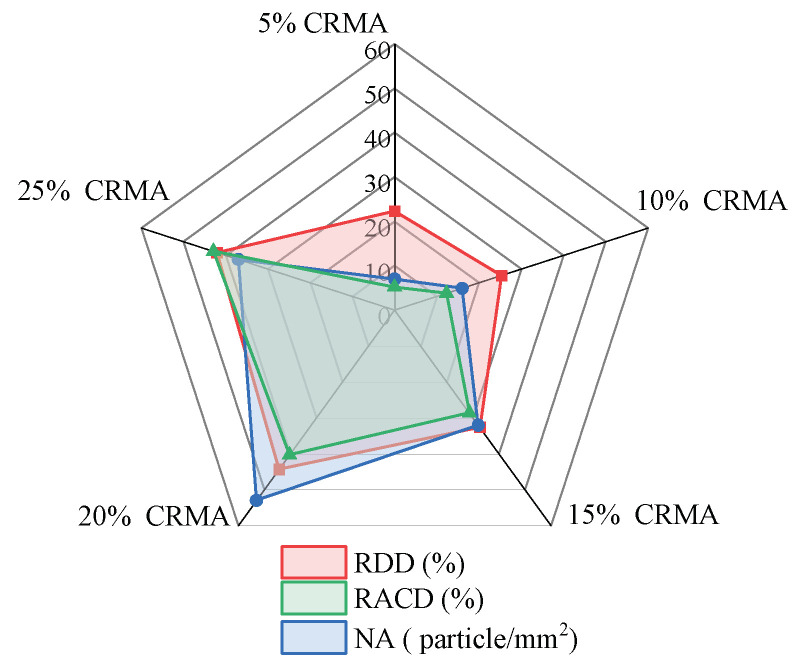
Changes in FM indicators of CRMA with different crumb rubber content.

The results of the FM analysis for CRMA, presented in Figure 9, include the RDD, RACD, and NA. As shown in Figure 9, as the crumb rubber content increased from 5% to 15%, NA increased significantly from 7 particles/mm^2^ to 32 particles/mm^2^, representing a 357.1% increase. Meanwhile, RDD rose from 22.3% to 32.6%, reflecting a notable increase of 46.2%. RACD expanded from 5.22% to 28.59%, marking a 447.7% increase. These results indicate that the newly added crumb rubber was primarily dispersed through the formation of new agglomerates, while both the existing and newly formed agglomerates underwent significant swelling and established a more complete interface with the base asphalt. At a crumb rubber content of 20%, NA increased sharply to 53 particles/mm^2^, representing a 65.6% rise compared to the 15% content. Similarly, RDD and RACD increased to 44.3% and 40.29%, respectively, showing additional gains of 35.9% and 40.9% compared to their values at 15% content. This suggests that, with good dispersion and adequate swelling, the crumb rubber began to form moderate internal connections, collectively contributing to the development of a well-structured three-dimensional network. However, when the content reached 25%, RDD decreased, NA reduced, and only RACD continued to increase. This indicates that the spatial distribution of the crumb rubber began to form larger and more concentrated dispersed-phase domains, which corresponds to the observed decline in macroscopic damping performance. The primary cause for this behavior is that the swelling capacity of the base asphalt likely approached saturation. Excessive crumb rubber resulted in a decrease in dispersion uniformity, disrupted the continuity of the network structure, and negatively affected the macroscopic properties.

**Figure 10:**
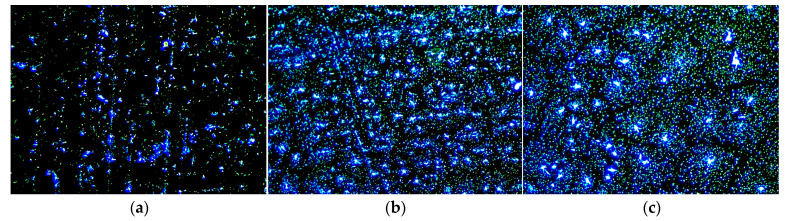
FM images of CRMA. (**a**) 15% CRMA; (**b**) 20% CRMA; (**c**) 25% CRMA.

To further validate the microstructural changes inferred from the aforementioned quantitative parameters, Figure 10 presents fluorescence micrographs of CRMA with crumb rubber contents of 15%, 20%, and 25%. As shown in Figure 10, when the crumb rubber content increased from 15% to 20%, the crumb rubber domains became more uniformly distributed and exhibited enhanced interconnectivity. This morphological evolution is consistent with the quantitative results, in which both RDD and RACD remained at relatively high levels, while NA increased significantly. Together, these indicators confirm the establishment of a moderately developed three-dimensional network. The DSR test results corroborate that the enlarged crumb rubber colloidal regions enhance viscoelastic energy dissipation, whereas the moderately interconnected three-dimensional network provides additional energy dissipation pathways as well as structural support. Together, these microstructural features synergistically contribute to the favorable loss modulus and damping efficiency observed for the 20% CRMA. However, when the crumb rubber content further increased to 25%, the fluorescence micrographs clearly show pronounced enlargement of the crumb rubber phase domains accompanied by localized enrichment, manifested as bright agglomerated regions. Consequently, this leads to a marked deterioration in the spatial uniformity of the crumb rubber distribution. These observations are in good agreement with the quantitative analysis: the reductions in RDD and NA correspond to the coarsening of the microstructure and a decrease in the number of agglomerates, while the increase in RACD reflects the expansion of individual agglomerate domains. Such microstructural degradation is associated with the decline in loss modulus and damping efficiency observed in the DSR tests for the 25% CRMA.

### 3.3. MD Simulation Results

#### 3.3.1. Solubility Parameter

The solubility parameter (*δ*_s_) is a crucial index for assessing the compatibility of materials. Its calculation is given by Equation (4):

(4) $$\delta_{s}=\sqrt{CED}=\sqrt{\frac{∆E}{V}}$$

where CED represents cohesive energy density, Δ*E* is cohesive energy, and *V* is volume.

CED quantifies the intermolecular interactions within materials. A lower CED indicates weaker intermolecular forces. According to the principle of similar compatibility, materials with similar polarity and smaller differences in solubility parameters tend to exhibit better compatibility [39].

After the dynamic calculation, the stable configuration from the final segment of the output trajectory file was selected as the basis for simulating the cohesive energy density. The solubility parameters were also directly simulated and obtained using Materials Studio. The solubility parameters of rubber and asphalt, along with their differences, are presented in Table 4.

As shown in Table 4, the *δ*_s_ value of the base asphalt is 18.87 (J/cm^3^)^0.5^, which aligns with typical asphalt values reported in the literature (18–20 (J/cm^3^)^0.5^) [37], thus confirming the reliability of the model. The solubility parameter analysis indicates that the |Δ*δ*_s_| values for all CRMA compositions are relatively small, suggesting good compatibility between the crumb rubber and base asphalt. As the crumb rubber content increases, the solubility parameter of the CRMA system gradually approaches that of the base asphalt. The minimum solubility parameter difference is observed at 20% and 25% crumb rubber content, indicating optimal compatibility between the two phases. Further analysis reveals that when the solubility parameters are well-matched, the effective penetration of asphalt’s lighter components into the crumb rubber network significantly reduces the system’s interfacial tension. This promotes better wetting and dispersion of the crumb rubber, which explains the observed improvements in crumb rubber dispersion and the expansion of the interfacial transition layer, as seen in the fluorescence microscopy images.

#### 3.3.2. Binding Energy

Binding energy is a key parameter used to measure the interaction energy between components in a mixed system. It can also serve as an indicator of the mixing capacity and compatibility between two materials within the system. Strictly speaking, the binding free energy comprises two components: enthalpy change and entropy change. However, in molecular simulations of complex systems, such as asphalt, directly and accurately calculating the entropy change is extremely challenging, as it requires determining the probability distribution of all microscopic states of the system [43]. As a result, the binding energy, as defined in Equation (5), which is the negative value of the system’s intermolecular interaction energy, is commonly used as a practical indicator for comparative analysis [39,42,44,45]. The calculation formula for binding energy is provided as follows:

(5) $$E_{binding}=-(E_{CRMA}-E_{asphalt}-E_{rubber})$$

where *E*_binding_ is the binding energy, *E*_CRMA_ is the total energy of the CRMA in its equilibrium state, and *E*_asphalt_ and *E*_rubber_ are the total energies of the asphalt and crumb rubber components in their respective equilibrium states.

**Figure 11:**
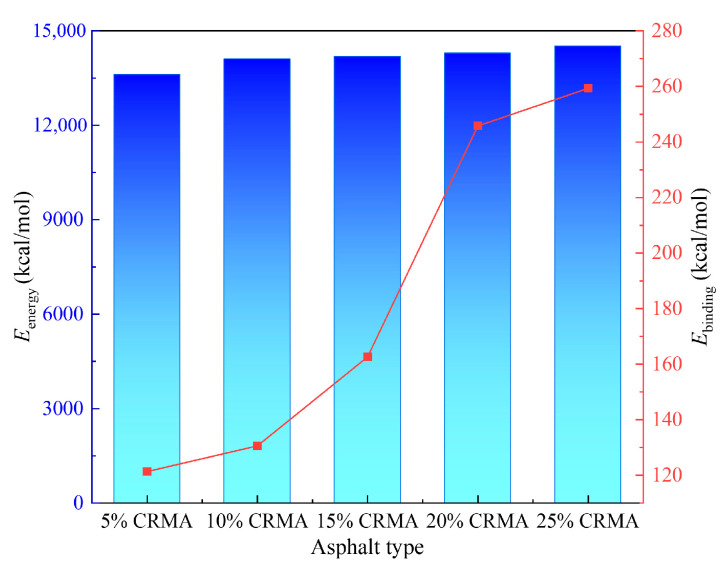
Binding energy of CRMA at different crumb rubber contents. The color gradient of the bars corresponds to the energy magnitude and does not convey additional quantitative information beyond the axis values.

The interaction becomes stronger as the binding energy increases, indicating higher thermodynamic stability and better compatibility between materials in the mixed system. The total energy and binding energy of CRMA are shown in Figure 11.

As shown in Figure 11, the total energy and binding energy of the CRMA system are significantly influenced by the crumb rubber content. As the crumb rubber content increases, the total energy of the system gradually rises, although the increase is relatively small. This suggests that the energy change in the system is primarily driven by the interfacial interactions between the crumb rubber and asphalt, rather than changes in the overall system energy. Moreover, as the crumb rubber content increases, the binding energy of the system also increases, with a notable transition occurring at 20% crumb rubber content. At this point, the binding energy increases by 88.2% compared to the 15% crumb rubber content, indicating a significant enhancement in the interfacial interaction between the crumb rubber and asphalt.

### 3.4. Quantitative Integration of Multi-Scale Parameters Using Gray Relational and Ridge Regression Analyses

To quantitatively elucidate the intrinsic relationships between macroscopic damping performance and micro/molecular parameters, and to develop a reliable prediction model, this study utilized gray relational analysis and ridge regression for multi-scale data fusion and modeling.

When constructing the multi-scale correlation model, it is crucial to select a macroscopic performance indicator that reliably characterizes the fundamental energy-dissipating nature of the material and demonstrates clear relationships with micro- and molecular-scale parameters. In this study, the loss modulus at 46 °C was chosen as the core macroscopic variable, based on three key considerations. First, the loss modulus directly represents the energy dissipated per unit strain due to viscous deformation, which is closely related to energy-dissipation mechanisms such as micro-friction, interfacial sliding, and molecular chain motion. This relationship facilitates the establishment of quantitative cross-scale connections [46]. Second, the overall stiffness of the asphalt mixture is primarily governed by the aggregate skeleton, while its energy dissipation capacity largely depends on the viscous behavior of the asphalt binder [3,30]. At the same time, Xu et al. [47] demonstrated that while the addition of crumb rubber reduces the binder’s loss factor by increasing its elasticity, it simultaneously enhances the binder’s loss modulus, which contributes to the significantly improved macroscopic damping performance of the rubberized asphalt mixture. Thus, the binder’s high loss modulus plays a pivotal role in determining the rubberized asphalt mixture’s damping performance. Third, as indicated by the data in Section 3.1, the loss modulus values of CRMA with different crumb rubber contents at 46 °C exhibit significant statistical distinction and show the most consistent variation trend. This allows for effective capture of macroscopic performance differences induced by microstructural changes, providing a high-discriminative power data foundation for subsequent modeling.

#### 3.4.1. Gray Relational Analysis

Gray relational analysis quantifies the degree of correlation between factors based on the similarity or dissimilarity of their developmental trends. It helps identify the key factors influencing the target value, thereby capturing the essential characteristics of the system. In this study, a gray relational evaluation model is developed using multi-scale data derived from DSR tests, FM observations, and MD simulations. This model is employed to quantitatively analyze the correlation between parameters at different scales and the damping performance of CRMA.

First, determine the reference sequence $x_{0}=\left\{\left.x_{0}(k)\right|k=1,2,\ldots ,n\right\}$, Then, establish the comparison sequence $x_{i}=\left\{\left.x_{i}(k)\right|k=1,2,\ldots ,n\right\}$ under the given conditions. The relationship between the reference sequence *x*_0_ and the comparison sequence *x_i_* can be expressed using the correlation coefficient:

(6) $$\varepsilon_{i}=\frac{m+\rho M}{{∆}_{i}(k)+\rho M}$$

where ${∆}_{i}(k)=\left|x_{0}(k)-x_{i}(k)\right|{,\;∆}_{i}(k)$ represents the difference sequence between the reference and comparison sequences. *M* and *m* represent the maximum and minimum values in the different sequences. *ρ* is the distinguishing coefficients, usually set to 0.5. Finally, the correlation degree is calculated:

(7) $$r_{i}=\frac{1}{n}\sum \varepsilon_{i}(k)$$

The parameters obtained from FM observations and MD simulations were used as the reference sequence, while the *G*″ values from the DSR tests served as the comparison sequence. The correlation between *G*″ and various parameters was analyzed using the gray relational analysis method. Python software (Version 3.6) was employed to calculate the correlation between *G*″ and the parameters. The results are presented in Table 5.

From Table 5, it is evident that there is a significant correlation between the microstructure parameters and the *G*″ of CRMA. Notably, the dispersion of crumb rubber exhibits the strongest correlation, highlighting its critical role in optimizing interfacial properties. Good dispersion not only increases the effective interface area but also enhances the efficiency of stress transfer. Additionally, the NA and RACD show strong correlations, underscoring the important regulatory role of crumb rubber phase morphology in influencing the damping performance of CRMA.

At the molecular scale, although the correlation between the solubility parameter and binding energy is relatively low, their values indirectly reflect the dual nature of the molecular mechanisms governing the damping performance of CRMA. In other words, optimal damping performance in CRMA requires a balance between intermolecular forces, which enhance energy dissipation, and compatibility, which ensures the stability of the interfacial structure.

#### 3.4.2. Ridge Regression Model

Ridge regression is a regularized regression technique designed to address collinearity in data. By introducing an L_2_ penalty term, it reduces model complexity, enhancing stability and generalization. This method is particularly effective in preventing overfitting, especially when dealing with small sample sizes, leading to more realistic and reliable regression coefficients with improved fitting performance. To develop a multi-scale quantitative prediction model for the damping performance of CRMA, this study uses the *G*″ as the response variable and applies ridge regression to systematically analyze the synergistic effects of microstructural parameters and molecular property parameters.

Before performing ridge regression analysis, it is essential to standardize the independent variables and assess collinearity. The standardization formula is as follows:

(8) $$x_{ij}^{\prime }=\frac{x_{ij}-\mu_{j}}{\sigma_{j}}$$

where *μ_j_* and *σ_j_* are the mean and standard deviation of the *j*-th variable, respectively. Standardization ensures comparability among variables with different units.

The formula for assessing multicollinearity of the standardized parameters is:

(9) $${VIF}_{j}=\frac{1}{1-R_{j}^{2}}$$

where VIF*_j_* represents the variance inflation factor for the *j*-th predictor, and $R_{j}^{2}$ is the coefficient of determination obtained from regressing the *j*-th predictor on all other predictors.

The results of the multicollinearity assessment are presented in Table 6. As shown, the VIF values for all parameters exceed 10, indicating the presence of significant collinearity in the data.

To construct a stable and interpretable optimal prediction model, variables were selected based on two criteria: the coefficient of determination (R^2^) from leave-one-out cross-validation and a VIF less than 5.

At the FM scale, five variable combination models were constructed based on the parameter importance ranking obtained from gray relational analysis: RDD, RDD + NA, RDD + RACD, NA + RACD, and RDD + NA + RACD. The evaluation results show that the simplest model, which includes only RDD, achieved a VIF of 1.00 and an R^2^ of 0.903. This demonstrates that the model not only avoids multicollinearity but also exhibits strong predictive accuracy and generalization capability. Consequently, this model was selected as the predictive model for the FM scale.

At the MD scale, the *E*_binding_ and the *δ*_s_ characterize the strength of intermolecular forces and thermodynamic compatibility, respectively, providing clear and complementary physical interpretations. By optimizing the regularization parameter of ridge regression, the collinearity between these two parameters was effectively suppressed, reducing their VIF to 4.02. Additionally, an R^2^ of 0.876 was achieved, indicating strong predictive accuracy.

**Figure 12:**
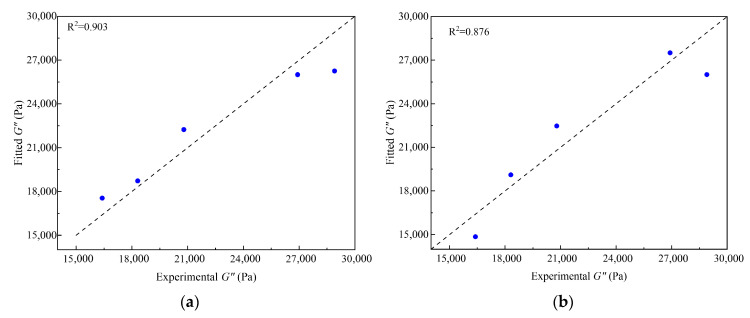
Experimental and fitted values comparison. (**a**) FM test; (**b**) MD simulation.

Based on the above analysis, ridge regression prediction models for the FM and MD scales were established (Equations (10) and (11), respectively). A comparison between the predicted and experimental values is shown in Figure 12.

FM-scale predictive model:

(10) G″=6748.16+430.73×RDD

MD-scale predictive model:

(11) $$G\prime \prime \;=45.82\times E_{binding}-19,693.30\times \delta_{s}+385,783.78$$

Figure 12 shows that the multi-scale prediction model established using ridge regression accurately predicts the loss modulus characteristics of CRMA. The model validation results indicate that the predicted data points are evenly distributed on both sides of the y = x reference line. Combined with the high goodness of fit, it can be concluded that the predicted values closely match the experimental values, demonstrating the strong reliability of the prediction model.

The FM-scale predictive model quantitatively characterizes the dominant influence of crumb rubber dispersion degree on the loss modulus. Its positive regression coefficient (430.73) indicates that a more uniform and refined dispersion of crumb rubber within the base asphalt significantly enhances damping performance, which aligns perfectly with the conclusions drawn from gray relational analysis. This enhancement is primarily due to the optimal dispersion state, which creates a larger interfacial area and more viscoelastic units, thereby facilitating more efficient energy dissipation through particle-matrix friction and hysteresis effects within the rubber-colloidal domains.

The MD-scale predictive model reveals the synergistic influence of molecular-scale parameters. The significantly positive coefficient of binding energy (+45.82) indicates that stronger intermolecular interactions between the crumb rubber and asphalt components promote energy dissipation. This aligns with the mechanism whereby enhanced interfacial adhesion facilitates stress transfer and molecular friction, leading to higher damping loss. In contrast, the solubility parameter exhibits a substantially negative coefficient (−19,693.30), which quantitatively characterizes a critical compatibility threshold within the CRMA system. Excessive mixing leads to an overly homogeneous molecular mix, forming a denser and more uniform network structure. This dense structure restricts the free movement of molecular chains and internal friction, thereby reducing the viscoelastic hysteresis necessary for achieving high damping performance. Therefore, a moderate degree of phase separation is beneficial for enhancing damping performance, which is consistent with the findings from the gray relational analysis.

## 4. Conclusions

DSR tests indicate that the damping performance of CRMA initially improves significantly with increasing crumb rubber content, before declining after a certain point, reaching optimal performance at 20% content. At this point, the loss modulus at 46 °C and 82 °C increases by 110% and 440%, respectively, compared to the base asphalt, and the damping-temperature spectrum is the flattest, with the highest loss factor value. This indicates that at this content, the material possesses excellent energy dissipation capacity and damping efficiency.

FM images and quantitative analysis further show that at crumb rubber contents up to 15%, the particles are well-dispersed, but have not yet formed an effective spatial network. At a crumb rubber content of 20%, NA increased sharply to 53 particles/mm^2^, representing a 65.6% rise compared to the 15% content. Similarly, RDD and RACD increased to 44.3% and 40.29%, respectively, showing additional gains of 35.9% and 40.9% compared to their values at 15% content. The morphology observed in the images confirms that, at this content, the crumb rubber undergoes uniform dispersion and sufficient swelling, resulting in the formation of a well-developed three-dimensional network structure.

MD simulation results show that as the crumb rubber content increases, the solubility parameter of the CRMA system gradually approaches that of the base asphalt, reaching its minimum at 20% content. At the same time, the interfacial binding energy experiences a significant increase (88.2% higher compared to 15% content), indicating that the system achieves optimal compatibility and the strongest interfacial interaction at this point, providing sufficient intermolecular forces for complete rubber swelling, uniform dispersion, and interface stability.

Gray relational analysis indicates that, at the micro-scale, RDD is the key factor most strongly associated with damping performance. At the molecular scale, the effects of interfacial binding energy and the solubility parameter on damping performance are comparable. To quantify these relationships, ridge regression was used to construct predictive models. The FM-scale model (R^2^ = 0.903) quantitatively confirms the dominant role of RDD at the micro-scale, while the MD-scale model (R^2^ = 0.876) reveals that damping performance is co-regulated and constrained by both interfacial binding energy and the solubility parameter.

The multi-scale correlation models and conclusions presented in this study are primarily based on the 90# Karamay asphalt and 80-mesh desulfurized crumb rubber used. While the models demonstrated high internal accuracy, their broader applicability requires future validation using a wider variety of raw materials. Nonetheless, the synergistic mechanism linking intermolecular interactions, micro-network structure, and macroscopic performance, which was revealed for the first time in this study, provides a theoretical framework and a quantitative research paradigm for a deeper understanding and precise regulation of CRMA’s damping performance.

## Figures and Tables

**Table 1:** Technical properties of base asphalt and crumb rubber.

Type of Material	Trial Items	Unit	Technical Requirements	Experimental Results	Specifications [24,25]
90# karamay base asphalt	Penetration	0.1 mm	80–100	81	JTG E20-2011 T0604
	Softening point	°C	≥44	45.5	JTG E20-2011 T0606
	Ductility 15 °C	cm	≥100	>100	JTG E20-2011 T0605
	Flash point	°C	≥245	309	JTG E20-2011 T0611
	Solubility	%	≥99.5	99.84	JTG E20-2011 T0607
80-mesh desulfurized crumb rubber	Relative density	g/cm^3^	1.10–1.30	1.17	JT/T 797-2019
	Moisture	%	<1	0.74	JT/T 797-2019
	Metal content	%	<0.05	0.041	JT/T 797-2019
	Fiber content	%	<1	0.71	JT/T 797-2019

**Table 2:** Molecular number and components of base asphalt.

Component of Asphalt	Molecular Number	Simulated Mass Fraction (%)	Experimental Mass Fraction (%)
asphaltenes	1	2.91	3.00
aromatics	38	22.74	22.47
resins	7	35.36	35.96
saturates	33	38.99	38.57

**Table 3:** The quantities of NR and SBR molecules at different crumb rubber contents.

Type	Number of NR Molecules	Number of SBR Molecules	Total Crumb Rubber Mass (g/mol)	Weight (%)
CRMA with a 5% crumb rubber content (5% CRMA)	2	1	1320.13	5.07
CRMA with a 10% crumb rubber content (10% CRMA)	4	2	2640.26	10.13
CRMA with a 15% crumb rubber content (15% CRMA)	6	3	3960.39	15.20
CRMA with a 20% crumb rubber content (20% CRMA)	8	4	5280.52	20.27
CRMA with a 25% crumb rubber content (25% CRMA)	10	5	6600.65	25.33

**Table 4:** Solubility parameters of CRMA at different crumb rubber contents.

Asphalt Type	CED (J/m^3^)	*δ*_s_ ((J/cm^3^)^0.5^)	|∆*δ*_s_| ((J/cm^3^)^0.5^)
Base asphalt	3.56 × 10^8^	18.87	
5% CRMA	3.64 × 10^8^	19.08	0.21
10% CRMA	3.61 × 10^8^	18.99	0.12
15% CRMA	3.58 × 10^8^	18.92	0.05
20% CRMA	3.55 × 10^8^	18.85	0.02
25% CRMA	3.57 × 10^8^	18.89	0.02

**Table 5:** Gray relational analysis of parameters and *G*″ values.

Parameters	FM Test			MD Simulation	
	RDD	RACD	NA	*E*_binding_	*δ*_s_
*r_i_*	0.7214	0.6483	0.6725	0.5729	0.5540

**Table 6:** The results of the multicollinearity assessment.

Parameters	FM Test			MD Simulation	
	RDD	RACD	NA	*E*_binding_	*δ*_s_
VIF	15.49	32.22	33.57	10.86	10.86

## Data Availability

Data is contained with the article.

## Abbreviations

The following abbreviations are used in this manuscript:

CRMA	Crumb rubber-modified asphalt
DSR	Dynamic shear rheometer
FM	Fluorescence microscopy
MD	Molecular dynamics
Tan *δ*	Loss factor
*G*”	Loss modulus
NR	Natural rubber
SBR	Styrene-butadiene rubber
RDD	Rubber dispersion degree
RACD	Relative area of colloidal domains
NA	Number of agglomerates per unit area
CED	Cohesive energy density
Δ*E*	Cohesive energy
*V*	Volume
*δ*_s_	Solubility parameter
*E*_binding_	binding energy
*E*_CRMA_	Total energy of the CRMA in its equilibrium state
*E*_asphalt_	Total energy of asphalt in its equilibrium state
*E*_rubber_	Total energy of crumb rubber in its equilibrium state
